# Differentiating multi-MeV, multi-ion spectra with CR-39 solid-state nuclear track detectors

**DOI:** 10.1038/s41598-023-45208-x

**Published:** 2023-10-24

**Authors:** M. S. Schollmeier, J. J. Bekx, J. Hartmann, E. Schork, M. Speicher, A. F. Brodersen, A. Fazzini, P. Fischer, E. Gaul, B. Gonzalez-Izquierdo, M. M. Günther, A. K. Härle, R. Hollinger, K. Kenney, J. Park, D. E. Rivas, V. Scutelnic, Z. Shpilman, S. Wang, J. J. Rocca, G. Korn

**Affiliations:** 1 Marvel Fusion GmbH, Theresienhöhe 12, 80339 Munich, Germany; 2 Electrical and Computer Engineering Department, Colorado State University, Fort Collins, CO 80523 USA; 3 Physics Department, Colorado State University, Fort Collins, CO 80523 USA

**Keywords:** Laser-produced plasmas, Plasma-based accelerators

## Abstract

The development of high intensity petawatt lasers has created new possibilities for ion acceleration and nuclear fusion using solid targets. In such laser-matter interaction, multiple ion species are accelerated with broad spectra up to hundreds of MeV. To measure ion yields and for species identification, CR-39 solid-state nuclear track detectors are frequently used. However, these detectors are limited in their applicability for multi-ion spectra differentiation as standard image recognition algorithms can lead to a misinterpretation of data, there is no unique relation between track diameter and particle energy, and there are overlapping pit diameter relationships for multiple particle species. In this report, we address these issues by first developing an algorithm to overcome user bias during image processing. Second, we use calibration of the detector response for protons, carbon and helium ions (alpha particles) from 0.1 to above 10 MeV and measurements of statistical energy loss fluctuations in a forward-fitting procedure utilizing multiple, differently filtered CR-39, altogether enabling high-sensitivity, multi-species particle spectroscopy. To validate this capability, we show that inferred CR-39 spectra match Thomson parabola ion spectrometer data from the same experiment. Filtered CR-39 spectrometers were used to detect, within a background of ~ 2 × 10^11^ sr^−1^ J^−1^ protons and carbons, (1.3 ± 0.7) × 10^8^ sr^−1^ J^−1^ alpha particles from laser-driven proton-boron fusion reactions.

## Introduction

Columbia Resin #39 (CR-39) is the trademark name of poly-allyl-diglycol-carbonate (C_12_H_18_O_7_), which has found numerous uses in a range of scientific and industrial applications. One of these uses is as a solid-state nuclear-track detector (SSNTD). Since its conceptual inception as a particle detector around sixty years ago^1–3^ it has been employed in medical research and biology^4, 5^, neutron dosimetry^6–8^, space physics^9–11^, nuclear physics^12–14^, laser-matter interactions and ion acceleration^15–25^, as well as in fusion experiments with deuterium–tritium^26–32^ or proton-boron fuels^33–42^, to name a few. In the demonstration of fusion energy output exceeding the laser energy on target (scientific breakeven or Q > 1) in December 2022^43^, CR-39 was used–among other detectors–to measure the fusion neutron yield at the National Ignition Facility (NIF)^7^. As an alternative to the indirect drive approach pursued at NIF, the development of short-pulse, high-intensity, petawatt lasers has opened new possibilities for initiating fusion reactions using advanced fuels such as proton-boron (pB)^42, 44^. Proton-boron fusion is an attractive fuel option for potential future commercial applications because the primary reactions are aneutronic and create solely three alpha particles per fusion event. Increasing numbers of alpha particles have been reported in the literature^33, 34, 36–42^ since the first observation of short-pulse laser-driven proton-boron fusion reactions by Belyaev in 2005^45, 46^. In all of those experiments, alpha particle detection was performed using CR-39 detectors due to their high sensitivity to single ions with nearly 100% detection efficiency, and their intrinsic calibration for absolute particle counts.

The operating principle of a CR-39 detector is rather straightforward. A particle striking the CR-39 plate deposits its energy by creating a proportional damage trail, referred to as a latent track, as it penetrates the detector. The geometry of this track depends on the incidence angle, energy and charge-to-mass ratio of the incident particle. The latent track is too small (few nanometers) to be observed optically but can be enlarged through chemical wet etching. Etching dissolves the polymer to the point where the track opening is large enough (few micrometers) to be observed and imaged by an optical microscope. In the microscope image, the track forms a round or elliptical pattern, referred to as a “pit”, that has brightness variation depending on the pit shape and depth. The number of particles originating from the source can be discerned using the total number of pits detected on the plate. Due to the high density of damage caused by energy deposition of ions as compared to electrons or photons, CR-39 is highly insensitive to electrons, electromagnetic pulses, x-ray, and gamma-ray irradiation.

Here, we show that arrays of multiple filtered CR-39 detector plates form a compact and inexpensive particle spectrometer that can be fielded in large quantities for three-dimensional, spatially resolved ion spectroscopy. The fastest, most-occurring accelerated ions in laser-matter interaction experiments are MeV-scale protons, e.g., from surface contamination layers, due to their lowest mass-to-charge ratio. Heavier ions such as carbon ions are accelerated to MeV energies as well. Particle spectroscopy with filtered CR-39 detector plates requires careful pit analysis, e.g., via numerical image processing of microscope data. Different particles of different energies might produce a pit of the same size, which requires calibration of the response of CR-39. In this context, we have performed a calibration for H, He and C ions with energies from hundreds of keV to several tens of MeV, including calibrations for the width of the distribution describing the ion energy loss statistics in the thin observation layer near the surface. After calibration, we present a forward calculation method using an assumed population of particle species such as protons and carbons or other ions to calculate the expected pit distribution in CR-39. Performing a Monte Carlo (MC) χ^2^ minimization of the input spectral parameters, a best match to the data is found. The forward calculation is performed simultaneously for several CR-39 plates in designed arrays, equipped with various filters to sample the spectra at multiple energy ranges of the spectral distribution. We show that the particle spectra for protons and carbon ions detected by CR-39 reproduce the spectra measured by a Thomson parabola ion spectrometer fielded in the same experiment.

### Unbiased pit recognition method

**Figure 1:**
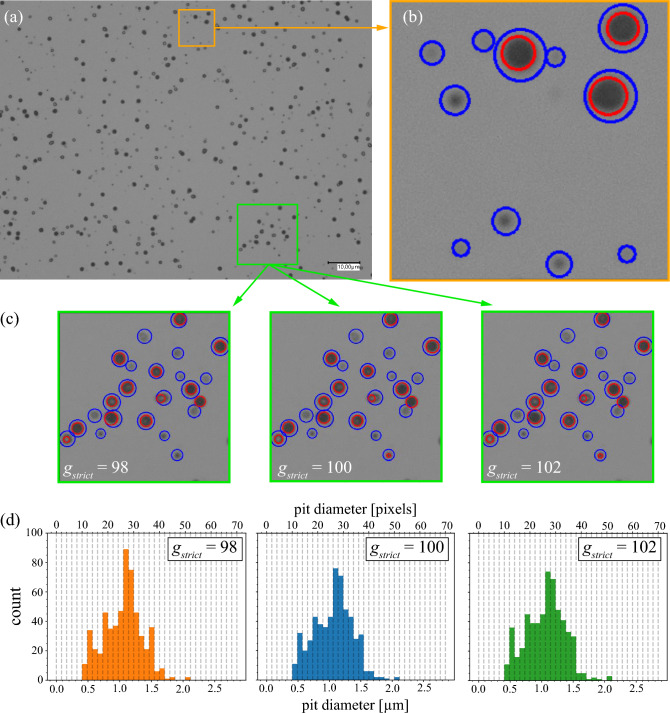
(**a**) Example of a grayscale image of a 114 µm × 85 µm section of an etched CR-39 plate. The orange and green frames denote sections of the image that are considered more closely for illustrative purposes. (**b**): Zoomed Sect. (10 μm × 10 μm) of the orange frame from panel a). The blue and red circles are the result of applying a Hough transform on the corresponding binary images with *g* = 132 and *g* = 100, respectively. The background had grayscale values around 137–142. (**c**): The zoomed in section (18 μm × 18 μm) of the green frame from panel (**a**). The blue and red circles depict the result of the Hough transform where the lax threshold is kept constant and the strict threshold parameter *g*_*strict*_ is varied slightly. (**d**) Histogram of number of pits found as a function of their diameter for the total CR-39 image depicted in (**a**) for the three thresholds. Visually, the variations in the strict threshold (panel **c**) do not differ all that much from one another, despite leading to inconsistent histograms depicted in panel (**d**).

After exposure to ions, the CR-39s are etched to enlarge the latent tracks and imaged with a high-resolution optical microscope (see example image in Fig. 1 and Methods). For each microscope image, the number of these track openings and their track parameters must be extracted. This is not a trivial task as evidenced by numerous publications^19, 31, 47–50^. Due to the large separation between source and detector in our experiments, the tracks are mostly circular where it suffices to measure the track diameter, which is the main parameter used in the remainder of this paper. A common approach to obtain the track diameter from an image is to use a Hough transform^19, 48^, for which one must first find the edges of the tracks. There are many different approaches to obtain edges in images, e.g., Sobel or Canny edge detection^51^. However, no matter which edge detection algorithm is employed, it will involve *thresholding* of some kind^19, 28, 39, 49, 50^. There is however an inherent issue with thresholding: it introduces a user bias on which grayscale value should be considered as an edge or not.

Consider the orange section of the example image, shown in Fig. 1a,b. It contains two families of circles, one faint and one pronounced. To distinguish the very faint pits from the background, one needs to employ a lax grayscale threshold value *g* for the Hough transform, resulting in the blue circles in Fig. 1b. While practically all pits were detected, this overestimates the track diameters, especially the more pronounced pits. More accurate pit diameters are obtained by using a stricter threshold (red circles), but then only three out of eleven pits are detected. Hence, a single threshold value does not in general allow for the determination of accurate track properties. One could introduce several threshold values, e.g., one for each level of “faintness”. But even this leads to inconsistent results because pits with a diameter that slightly exceed the threshold will be grouped together as having the same diameter. This is illustrated in Fig. 1c,d. Pits are first found with a lax threshold (*g*_*lax*_, blue), and those that are also found with a strict threshold *g*_*strict*_ are overwritten (red). Visually, the variations in *g*_*strict*_ (Fig. 1c) do not differ much from one another. However, even slight variations of *g*_*strict*_ lead to very different statistical behaviors emerging in the diameter histograms shown in Fig. 1d. For example, the pronounced peak at *D* ≈ 1.5 μm for *g*_*strict*_ = 98 completely vanishes for *g*_*strict*_ = 102. Also note the sudden drop-off at *D* ≈ 1.5 μm for all histograms, which is an artifact arising from the fact that pits with diameters slightly above this value are erroneously reduced by the imposed strict threshold, causing the corresponding bin at *D* ≈ 1.5 μm to be artificially inflated. This is the essence of the problem with thresholding: It is an inherent user-dependent input, which leads to the possible emergence or disappearance of peaks in the pit size distributions depending on user-selected thresholds.

It is of note that the user bias issue is particularly important if the pits are small with diameters nearing the resolution limit of the microscope. The finite optical resolution leads to softening of the edges of the pit image, making it particularly susceptible to thresholding issues. Longer etching times produce more pronounced pit edges compared to the microscope resolution, reducing the threshold issue. However, this may not always be an option as it eventually leads to track overlap or to the emergence of previously unobserved tracks such as from high-energy protons^18, 38^. Therefore, short etching times are favorable to combat a possible oversaturation and track overlap in the detector.

The Hough transform may have difficulties in obtaining the pit diameters in a consistent manner, but it is quite adept at finding the pit centers and is used by us for this purpose. For the pit diameter, we have developed a method referred to as the “Half-width-at-half-maximum (HWHM) Method” applying the following reasoning. As one traces a lineout through the center of an idealized, circular pit opening, the encountered grayscale values will gradually decrease from background while crossing the pit boundary until a minimum is reached at the center. Continuing along the lineout back to background, we expect a symmetrically mirrored behavior. Such behavior can be modeled and fitted analytically to achieve a threshold-free criterion for the pit diameter. For sharp pit edges, the gradient is given by the point spread function of the microscope, which typically is described by an Airy function. For a simpler analytic treatment, we take a Gaussian:

1 $$f(x) = A\left[ {1 - e^{{ - \frac{1}{2}\left( {\frac{x - \mu }{\sigma }} \right)^{2} }} } \right] + B,$$

where *B* is the minimal height of the function at *x* = μ, *A* + *B* is the height of the tail of the function, and μ and σ^2^ are the conventional mean and variance. In a realistic scenario, the pixels will not perfectly follow a Gaussian behavior. Some brighter pixels may show up near the center. To mitigate this noise, a radial averaging is taken instead of taking a lineout. Furthermore, the center pixel is not always the darkest one for any given pit. This offset needs to be accounted for by allowing |µ| to deviate from zero, resulting in a bias-free definition for the pit radius by assigning it to the HWHM of the Gaussian: r_HWHM_ =|µ|+ (2 ln2)^1/2^ σ, where σ > 0. The efficacy of this method is illustrated in Fig. S2 ﻿in the Suppl. Materials. Note that the HWHM-radius, as it arises from the fitting process, can take on rational values, despite it being represented in unit of pixels. This enables *super-resolution binning* in subsequent count histograms. We note that HWHM is not the only possible criterion and other criteria may be applied (see Suppl. Materials), so long as it is consistently used in both calibration and experimental measurements.

**Figure 2:**
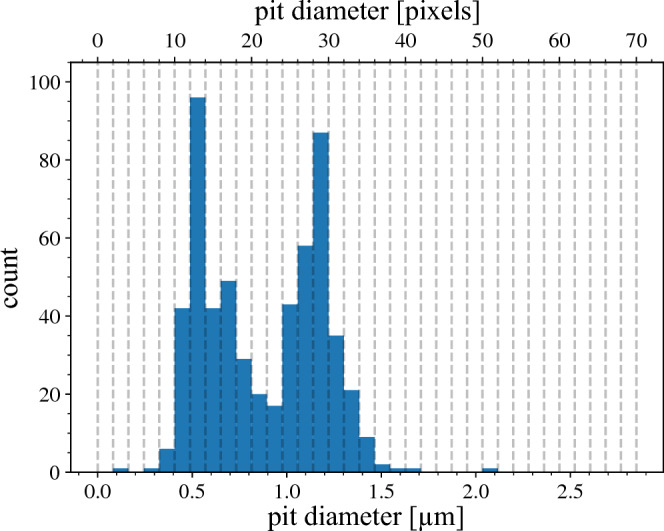
Histogram of number of pits found as a function of their diameter for the total CR-39 image depicted in Fig. 1a using the HWHM-method. In contrast to Fig. 1d, the HWHM method results in two distinct peaks at around 0.5 and 1.2 µm, in striking contrast to any of the previous histograms in Fig. 1c, as well as the absence of the artificial cut-off at around *D* ≈ 1.5 μm.

Finally, we apply the HWHM method to all pits in the original CR-39 image in Fig. 1a, resulting in the histogram shown in Fig. 2, which is strikingly different and shows two distinct peaks instead of the irregular, broad distribution. The two peaks indicate that two different particle species impacted on this CR-39. This result demonstrates that edge detection algorithms are very susceptible to user defined parameters and lead to incorrect results for CR-39 analysis conditions like those used here. The HWHM method removes this issue.

### Calibration of pit diameter versus particle species and energy

**Figure 3:**
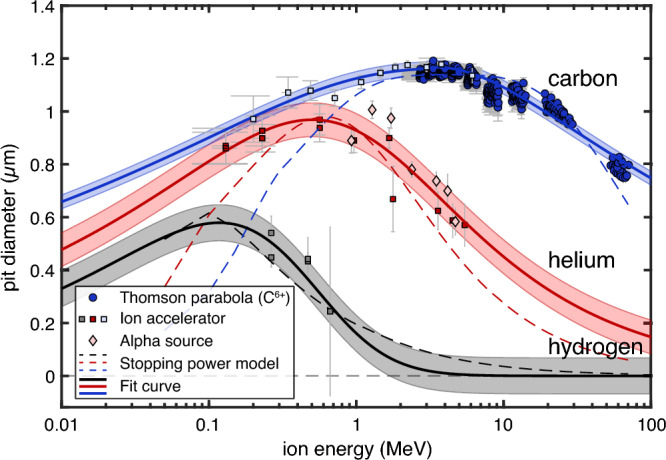
CR-39 calibration curves for protons (gray), helium ions (red) and carbon (C^6+^, blue) from 0.1 MeV to 70 MeV ion energy, after 30 min of etching in 6.25 M NaOH at 70 ℃. Note the logarithmic abscissa. The symbols depict the most probable diameters and the error bars the ± σ-widths of the pit diameter distribution for each data point. Calibration data points obtained at the ion accelerator are plotted with squares, carbon results from the Thomson parabola ion spectrometer are plotted with circles, and He ion (alpha particle) data from a ^239^Pu source are plotted with diamonds. The dashed lines show the scaling of deposited energy within 2 µm of CR-39 using a stopping power model based on SRIM data. Since the stopping power model deviates towards both high and low energy limits of the data, we used an analytic fit (Eq. 2, continuous lines) for a better match. As a measure of the error of the fit, the shaded bands represent the variance (± 2.35σ) of the residuals between measured data and the fit. The fit parameters and variance values are given in Table 1.

For the calibration of pit diameter versus particle species and their energy, we combined data obtained at a tandem accelerator, from a Thomson parabola ion spectrometer (TP), and from an alpha emitter source (see Methods for details). The irradiated CR-39s were processed via the HWHM method and analyzed for the most probable pit diameter and the width of the distribution (see next section). The results are plotted in Fig. 3. Most strikingly, the calibration curves are not bijective, i.e., for a given pit diameter there are two possible energy values even for single-species irradiation. Even though there are similar results in the literature^18, 20, 29, 45, 52–54^, the non-bijective nature for particle identification has not been discussed. Consequently, a particle cannot be identified by pit diameter alone. For example, a pit diameter of 0.8 μm could have been created by a helium ion with about 0.2 MeV or 2 MeV, or by a carbon ion with about 0.1 MeV or about 70 MeV. Furthermore, the curves partly overlap within their error bars for some energies, e.g., carbon ions and alpha particles between 0.1 and 0.4 MeV. When applied to the measurement of few-MeV helium ions from proton-boron fusion, where the expected pit diameters are at around 0.6 μm (~3-5 MeV) in our case, we find an overlap with the peak of the proton curve at 0.1–0.2 MeV. Longer etching may further separate the curves from each other^53, 54^, but it will not resolve the non-bijectivity.

The pit diameters are proportional to the stopping power *dE*/*dx* of the incoming particle. As shown in Fig. 3, we obtain a decent match to the data when using a SRIM^55^ data table to calculate the energy deposition in the first 2 µm of CR-39. This was obtained by artificially constraining the stopping power *dE*/*dx* to an upper limit during the tracking of the ion in the material and including a smooth transition to this limit. The common justification is that a high *dE*/*dx* leads to the generation of more secondary δ-electrons that transport a fraction of the energy out of the track volume and thus do not contribute to track formation^18^.

While the stopping power model provides insights into the shape of the calibration curves, the fit to the data is not satisfactory. A better match was obtained by fitting an analytic function to the data. Here, a modified beta distribution was chosen:

2 $$y(x) = \left[ {\frac{{p_{1} x^{{p_{2} - 1}} }}{{(1 + x)^{{p_{2} + p_{3} }} \beta (p2,p3)}}} \right]^{{p_{4} }} ,$$

where β = Γ(*p*_2_)Γ(*p*_3_)/Γ(*p*_2_ + *p*_3_), Γ the gamma function, and *p*_1,2,3,4_ are the fit parameters. Table 1 summarizes the fit parameters for the three ion species as well as the variance (± 2.35σ) of the residuals between measured data and the fit.

**Table 1:** Fit parameters for the calibration curves in Fig. 3 and standard deviation σ of the residuals of the fits.

Ion	p_1_	p_2_	p_3_	p_4_	σ (µm)
H	4.40	2.5	1.15	0.28	0.058
He	47.83	0.49	1.53	0.05	0.054
C < 3 MeV	7459.8	0.03	5.68	0.23	0.025
C ≥ 3 MeV	70.95	0.10	7.99	0.96	0.025

### Energy loss statistics in the thin surface observation layer of CR-39

Our etching procedure for the CR-39 results in pits of ~ 1 μm diameter and ~ 1 μm depth. The stopping range of MeV-scale ions is 2–10 times longer than this depth. This means the etching only reveals the energy deposited within the first few microns, rendering the CR-39 equivalent to a very thin detector layer. For thin detectors, the energy loss probability distribution is described by the highly skewed Landau (or Landau-Vavilov) distribution^56^, which provides a statistical description of the most probable energy loss *µ*_L_ and the width of the distribution *σ*_L_. It is important to note that while the Landau theory provides a good description of the energy loss fluctuations for relativistic particles, it may not be accurate for slower particles where additional effects, such as electron–electron interactions or lattice effects, become more significant. In those cases, other models^57^ or MC simulations^55^ may be more appropriate. An effective description is captured by convolving the Landau distribution with a Gaussian, referred to as ‘Langau’^58^, which adds another parameter *η*_L_ to the distribution that describes the Gaussian width.

**Figure 4:**
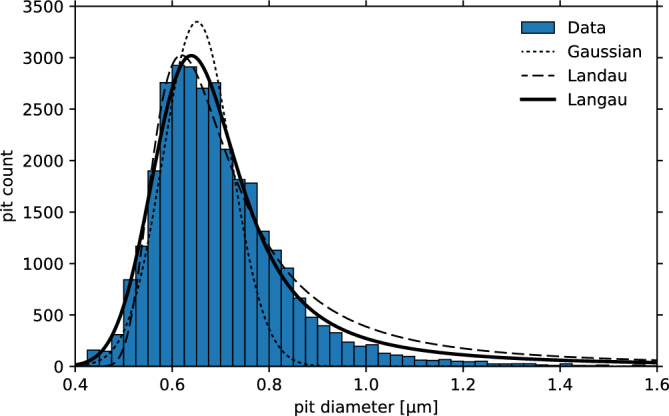
Distribution of pit diameters after (2.25 ± 0.06) MeV He ion irradiation. He ions with this energy have a stopping range of 9.91 µm according to SRIM, which is well above the pit depth expected for our etch conditions. The distribution exhibits a tail towards larger pit diameters, which can be well described by a Langau distribution centered at µ_L_ = 0.639 µm with σ_L_ = 0.059 µm and η_L_ = 0.035 µm (continuous line). A pure Gaussian/Landau (dotted/dashed lines) distribution would underestimate/overestimate the tail.

Figure 4 shows that a Langau distribution accurately describes the measured pit size distribution. Note that similar distributions for short etch times have been published earlier, e.g.,^19–21^, without recognizing their importance for particle identification. A proper inclusion of the Landau or Langau distribution is crucial for a correct interpretation of experiment data from a laser particle acceleration experiment. Figure 4, as well as Figure S3 of the Supplements, in combination with the calibration data in Fig. 3, show that the Landau tails lead to a strong overlap of the pit diameters for particles of interest, and particularly for alpha particles when protons are present. Crucially, depending on the relative particle flux and overall signal-to-noise level of the data, the tail of the skewed proton distribution may be interpreted as an alpha particle signal, as is shown in the Suppl. Materials.

**Figure 5:**
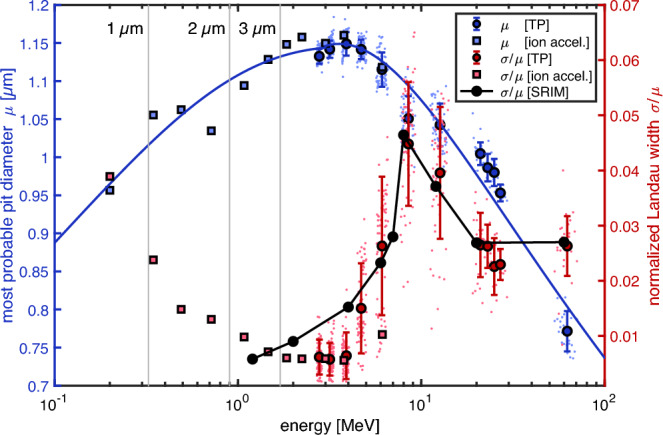
Most probable pit diameter μ (blue) and normalized Langau width σ = σ_L_ + η_L_ (red) for carbon ions versus ion energy. Data from the ion accelerator calibration are plotted as squares. Data from the TP analysis are plotted as dots marking the width of histograms from single microscope images. The larger circles plot the average width, the error bars denote the variance of the data. The continuous blue line is the calibration curve discussed in Fig. 3. The scaling of the relative width of the Langau distribution is non-monotonic and different from the scaling of the most probable pit diameter. The width of the Langau distribution was divided by μ to show the relative scaling in a unitless quantity. In black we plot scaled results of a TRIM simulation for the vacancy production in the first µm of CR-39, which exhibits a similar scaling. The gray vertical lines mark the initial energy of C ions corresponding to a 1, 2, or 3 µm stopping range, which is close to the etched pit depth. Near the end of the ion range, the distribution broadens and can no longer be described by a Langau distribution.

To understand how these distributions vary with ion energy, Fig. 5 shows the scaling of the normalized Langau distribution width *σ*/*μ* = *(σ*_*L*_ + *η*_*L*_*)/μ* versus energy. The maximum width is ~ 0.05 for an energy of ~ 10 MeV. This maximum does not coincide with the maximum pit diameter, which is observed to be at 4 MeV. After passing through a minimum at ~ 2–6 MeV, the width of the distribution strongly increases for lower energies. A similar behavior was observed for the He ions (not shown) but shifted towards lower energy values and higher σ/μ values. The increasing σ/μ for energies below 1–2 MeV may be explained by the fact that carbon ions at these energies are fully stopped within a few microns range. The gray vertical bars mark the incoming carbon energy for a range of 1, 2, and 3 μm. Our etch conditions result in a track depth of ~ 1–2 μm, which means that for the low energies the etching has already moved near or beyond the stopping range, where energy straggling leads to a significant broadening of the pit size distribution^18^.

For further insights into the scaling of the observed width of the pit distribution for energies above one MeV, we have performed ion tracking simulations with TRIM^55^ analyzing the vacancy production to quantify the radiation damage^59^. We simulated the depth-dependent vacancies produced by 2,000 incident ions within a volume of zero to 1 μm of CR-39. Assuming a direct proportionality between the vacancy count and pit diameter in CR-39, we retrieve the most probable vacancy count μ_TRIM_ and width σ_TRIM_ of the distribution (see Suppl. Materials for details). The TRIM results were scaled with a constant factor to convert from vacancy number to pit size. Additionally, the energy values had to be scaled by a factor of ~ 2 to get a better match to our measurements. The shift in projectile energy may be due to different material densities between simulation and experiment, or different observation volumes. The important message is that the well-tested vacancy production model of TRIM shows the same scaling with energy as our measurements, explaining the observed pit size distributions for monoenergetic ions.

### Forward fitting methodology

Having understood the CR-39 response to individual ions, we now introduce a method to analyze pit size distributions for tens of thousands of pits from laser-matter interaction experiments to obtain multi-particle spectra. CR-39 plates are equipped with multiple adjacent filter foils of different thickness and material, which prevent ions stopped within the foil from creating a track in CR-39. Due to the different stopping powers of particles of different mass within the filters it becomes possible to obtain more information about the respective particle energy spectrum. As shown above, simplistic pit size measurements will lead to incorrect particle numbers and their spectra due to significant energy loss statistics and partial overlap of the calibration curves. Here, we propose a refined analysis method, which is based on adding prior knowledge about particle distributions in a self-consistent fashion. Our method was independently developed but turned out to be similar to a method used to infer fusion proton and deuteron spectra in implosion experiments from filtered CR-39 plates^60^. We start with the prior knowledge that laser-driven ion emission spectra from solid targets are almost always exponentially decaying^61–64^. Most spectra can be described by a Boltzmann distribution:

3 $$\frac{dN}{{dE}} = \frac{{N_{0} }}{E}{\text{exp}}\left( { - \frac{E}{{k_{B} T}}} \right),$$

or a modification thereof in case of an isothermal plasma expansion^65^:

4 $$\frac{dN}{{dE}} = \frac{{N_{0} }}{{\left[ {2Ek_{B} T} \right]^{1/2} }}\exp \left( { - \sqrt {\frac{2E}{{k_{B} T}}} } \right),$$

where *N*_0_ is the total particle yield and *k*_*B*_*T* describes the slope (or temperature) of the spectrum. This leads to the following observations and conclusions for fitted CR-39 measurements:

1. Filter foils in front of CR-39 act like a high-pass filter: all ions with energies significantly above the filter threshold are transmitted with negligible energy loss, while ions below the threshold are blocked. Near the threshold the energy loss is no longer negligible. We use SRIM data tables to accurately track the energy loss of ions propagating through the filters.﻿
2. Exponential particle spectra before the filter are still exponential after the filter, resulting in a broad distribution of pits for each particle species. The measured pit sizes can be described by the product of the energy spectrum after the filter times the calibration curve.
3. For any given ion at any given energy, the energy loss probability is described by a Langau distribution. Therefore, the measured pit size distribution is the result not only of the particle spectra times the calibration curve, but also of the convolution with a Langau distribution corresponding to the particle energy and species.
4. Instead of unfolding the spectra from measured pit size distributions, which is not trivial due to the strong non-linearities and noise involved, we perform a forward-fitting procedure: Starting with an assumption of the spectral distribution such as Eq. (3) or Eq. (4)﻿ we generate a calculated pit size distribution for protons, heavy ions (carbons), and alphas each, then add the pit size distributions to a combined histogram and compare this distribution to the measured one:
  5 $$\frac{dN}{{dr}} = \mathop \sum \limits_{i = 1}^{n} \left( {\frac{dN}{{dE}} \times \frac{dE}{{dr}}} \right)*L\left( r \right),$$
  where a particle spectrum *dN*/*dE* is first multiplied by the distribution of pit diameter 2*r* versus energy *dE*/d*r* (Eq. 2), and the resulting product is then convoluted with a Landau distribution *L*(r) to include the energy loss probability statistics. The sum is taken over all particle species, e.g., protons, carbon (or boron), and alpha particles.
5. Such a forward calculation is performed simultaneously for several CR-39 data, which were all filtered differently and placed next to each other in an experiment, instead of just one filtered CR-39, to be much more sensitive to the spectral shape.﻿
6. A best fit is obtained by minimizing a modified, logarithmic χ^2^ criterion:
  6 $$\chi_{j} = ln \left[ {1 + \frac{{\mathop \sum \nolimits_{{i = D_{min} }}^{{D_{max} }} \left( {\text{measured}_{i} - \text{calc}_{i} } \right)^{2} }}{{\mathop \sum \nolimits_{{i = D_{min} }}^{{D_{max} }} \text{calc}_{i} }}} \right],$$
  w﻿here χ_*j*_ for one filtered CR-39 histogram *j* is calculated as the squared difference between measured and calculated counts, summed over all diameters from a user-chosen minimum diameter *D*_min_ to the maximum diameter *D*_max_, and normalized by the total calculated counts as a weighting factor. The + 1 is needed to avoid undefined behavior. The total difference χ for all CR-39 forming a common detector is calculated as the sum of squares of the individual χ_j_.

By doing so, the ion spectra parameters (*N*_0_, *k*_*B*_*T*) that best fit the measurements can be selected. Our numerical implementation of the forward fit additionally includes compensating for potential etch variations between the different CR-39, as well as compensating for potential particle counting fluctuations compared to the analytic distribution. In the Suppl. Materials, we present artificial pit size distributions calculated by the forward fitting method and, using artificial data with added noise, we verify that the presented minimization method reproduces the true spectra. In the case of a small population of alpha particles compared to protons or carbons, we show that this population can be retrieved with reasonable error bars.

## Experimental validation

The CR-39 spectrometer was used in an experiment campaign at the Laboratory for Advanced Lasers and Extreme Photonics at Colorado State University, Ft. Collins, CO, USA. T﻿he 80-fs, 3-J laser pulse was used to irradiate a commercial, 2-mm-thick boron sample. Ions were detected with two sets of filtered CR-39 spectrometers. Further details about the experiment are listed in the Methods sections.

**Figure 6:**
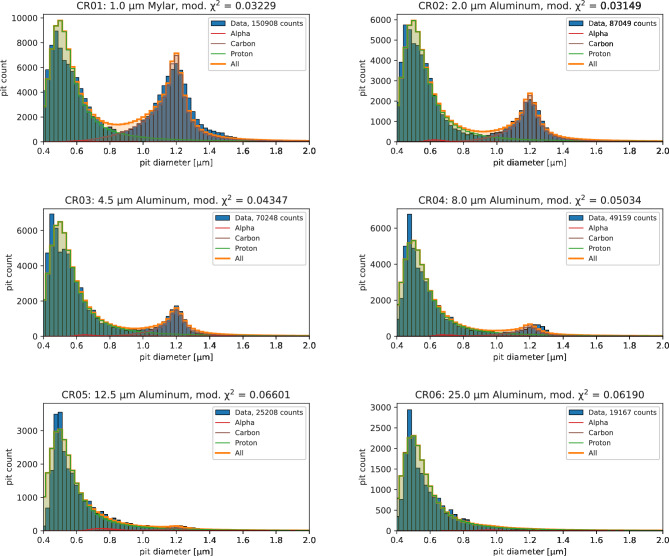
Analysis of data from an experiment using a high-intensity short-pulse laser to irradiate a commercial boron plate. Each plot (blue bars) represents the pit size distribution for an array of six filtered CR-39. The header notes the filter material and thickness through which the particles had to traverse before impacting the CR-39. It also notes the minimum χ^2^ value for each plot. The orange line plots the resulting best-fitting calculated histogram, consisting mainly of protons (green line), carbon or boron ions (brown line), and a small fraction of alpha particles (red line).

Figure 6 shows the counts-versus-diameter histograms for an array of six filtered CR-39 compared to the calculated, best-fitting artificial histograms. The histograms were generated by analyzing 100 microscope images for each filter, comprising a total of 600 CR-39 images. Each measured CR-39 histogram had to be shifted by a few percent (typically below ± 3%) along the diameter axis to achieve the best-possible fit. Additionally, in all the CR-39 from this experiment the proton calibration curve must be shifted by about − 15% with respect to the calibration curve plotted in Fig. 3, indicating that the calibration data were insufficient to determine the peak location of the hydrogen curve.

Most particles detected in this experiment are protons, with a most probable diameter of ~ 0.5 µm, and carbon or boron ions with a most probable diameter of ~ 1.2 µm. CR-39 calibration for boron ion was not possible with the data from this campaign. Since the charge-to-mass ratio of boron ions is similar to carbon ions, we expect the two ion species to create very similar pit diameter distributions and consider them virtually indistinguishable. The right tails of both peaks in each CR-39 are mainly due to energy loss statistics (Langau distributions). A good match to the data was obtained using σ_L,p_ = 0.035 μm, *η*_L,p_ = 0.015 µm for the protons and σ_L,c_ = 0.025 μm, *η*_L,c_ = 0.009 µm for carbons, in agreement with the expected data for low-energy particles (see Table S1 in the Suppl. Materials) .

After manually finding an initial, close match to the data, a MC scan was performed for 50,000 parameter samples, randomly varying the analytic spectra parameters from 0.5 to two times the manually found optimum to find the global minimum. Such a sensitivity scan is used to not only infer the best-fitting parameters but also the error of the fit due to the noisy input. The MC results were afterwards filtered by only those parameters that are within ± 25% of the global minimum. The global minimum was found for *N*_0_ = 1 × 10^11^ sr^−1^ J^−1^ and *k*_*B*_*T* = 4.36 MeV for protons with a spectrum described by Eq. (4), and *N*_0_ = 1.4 × 10^10^ sr^−1^ J^−1^ and *k*_*B*_*T* = 2.01 MeV for carbons with a distribution described by Eq. (3).

**Figure 7:**
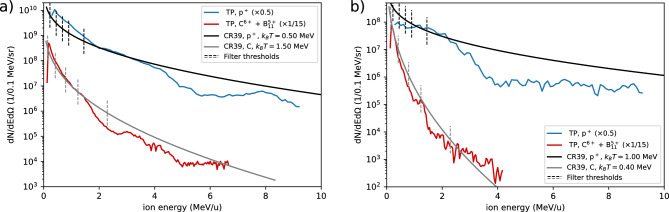
Comparison of ion spectra measured by the Thomson parabola (TP) ion spectrometer compared to the CR-39 method. (**a**) shows the measurement described in text. In (**b**) we show results from a different sample to demonstrate the reliability of the method in inferring particle spectra. The best-fitting spectra for the CR-39 are similar to those measured by the TP, demonstrating the validity of the method. The dashed, vertical lines mark the filter thresholds of the used mylar and aluminum filters (see Fig. 6 for details), which were tuned for few-MeV heavy ions.

Analysis of the second CR-39 array of the same experiment (CR39a, see Methods) results in 1.6 × 10^11^ sr^−1^ J^−1^ protons with *k*_*B*_*T* = 0.5 MeV and 3.4 × 10^10^ sr^−1^ J^−1^ carbon ions with *k*_*B*_*T* = 1.5 MeV. For a demonstration of the validity of the forward-fitting method, we compare the spectra obtained by this second CR-39 array to measurements obtained by the Thomson parabola (TP) ion spectrometer^66^, since the CR-39 array was placed at the vacuum port closest to the TP. Figure 7 shows the spectra measured by the TP, compared to the spectra obtained by the CR-39 array, for two different targets. Both the carbon and proton spectra are well reproduced, except for a constant multiplier in the absolute particle flux and some spectral modulations. The CR-39 spectrometer measured twice as many protons as the TP. Note that the scanner used to scan the Fuji BAS-TR image plate fielded in the TP was only coarsely calibrated for protons in an earlier experiment^67^, which can explain the factor of two difference between TP and CR-39 data found here. Due to a lack of carbon ion calibration of the used scanner, we used a calibration by Doria et al.^68^ for a different scanner to convert from image plate units to particles. We therefore expect differences in absolute numbers between the ion spectra obtained by the TP compared to CR-39.

Since protons from the contamination surface layer may be accelerated enough to trigger proton-boron fusion reactions that result in alpha particles, we included a Gaussian distribution of alpha particles in the forward fit and the MC scan.

**Figure 8:**
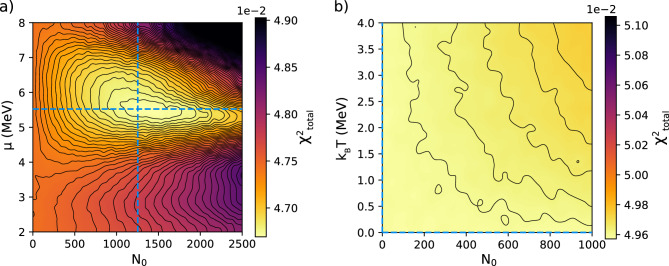
Sensitivity scan for alpha particles. The figure shows the variation of the total *χ*^*2*^ value for all six filtered CR-39 used in the array, when the assumed number of alpha particles *N*_*0*_ and their average energy *µ* or temperature *k*_*B*_*T* are varied. In (**a**), we assumed a Gaussian spectrum, characterized by *N*_*0*_ and *µ*, with a full-width-at-half maximum of 1 MeV. To generate this plot, the input parameters for all three particles were sampled 50,000 times with a MC method and afterwards filtered by only those parameters that are within ± 25% of the global minimum. The MC scan reveals a global optimum, marked by the dashed lines. In (**b**), we show results of a second MC scan assuming an exponential distribution, characterized by *N*_*0*_ and *k*_*B*_*T*, described by Eq. (4), instead of a Gaussian, which results in a global optimum at zero.

In Fig. 8 we show two-dimensional maps of the total *χ*^2^ versus particle number *N*_*0*_ and average energy µ for the alpha particles within the data plotted in Fig. 6 for CR39b. We find a global optimum at *N*_0_ = 1253 and *μ* = 5.5 MeV for the alpha particles, corresponding to ~ 200 detected alpha particles per shot. A second MC scan assuming an exponential particle spectrum instead of a Gaussian leads to an optimum at zero. This result is reassuring in that it shows that there is a unique solution for the alpha particle spectrum. Analysis of the second CR-39 array (CR39a, Fig. 7a) of the same experiment results in ~ 200 alpha particles per shot as well, but centered at a slightly higher energy of 6.1 MeV.

The error of the resulting parameters is mainly determined by the particle counting statistics given by $\sqrt{N}$, the variance of the calibration data given in Table 1, the error of the best-fitting width of the Langau distribution (which is determined by analyzing the measured tails of the histogram), the error of the etch process (e.g., duration and temperature), and image acquisition error (e.g., spatial resolution, focusing error due to sample homogeneity and surface variation), both of which are determined by the required diameter adjustment of the measured versus calculated histograms, and the error or sensitivity of the fitting method itself. The latter has, due to the low relative alpha particle yield compared to protons or carbons, the largest contribution to the overall error. The color gradient in Fig. 8 shows that the fit is much more sensitive to changes of µ_0_ than *N*_0_. A change of the mean energy corresponds to a change of the alpha pit diameter histogram towards larger or smaller values. The He ion calibration curve has a steep slope at around 5 MeV, which explains the strong sensitivity of the fit to this parameter. A change in the particle number increases or decreases the amplitude of the histogram, which, due to the low alpha count compared to protons or carbons, is less sensitive to changes and more susceptible to noise. We assign the full-width-at-half-maximum (FWHM) of the gradients along the dashed lines as a measure of the fit error. After Gaussian error propagation, we determine the global optimum for the alpha particles as *N*_0_ = 200 ± 110 (ΔN_0_/N_0_ = 56%) per J per shot for both CR39 detectors and average energies of *μ*_*CR39a*_ = (6.1 ± 0.6) MeV and *μ*_*CR39b*_ = (5.5 ± 1.4) MeV.

The average energy for the He ions is within the range of expected energies from proton-boron fusion reaction kinematics when the protons are accelerated to about 1 MeV energy^46, 69, 70^. Such a proton energy is near the peak of the proton-boron fusion cross section and is also the most abundant energy in the TP spectrum (Fig. 7). The fact that the alpha particles detected by CR39a, which was placed closer to the target normal, are slightly higher than those detected by CR39b might be explained by particle acceleration due to electrostatic sheath fields near the surface^38^.

To further investigate the detection limit of this technique of inferring alpha particles, we performed the experiment on a target where no significant alpha particle emission is expected. For this, in a second experiment, we used a pure graphite plate instead of boron as a target. Performing the same analysis as above results in an eight times lower proton count per Joule of laser energy but a 24 times lower alpha particle yield at a similar energy as for the boron plate, further confirming that the boron plate irradiation produced measurable alpha particles. The non-vanishing alpha particle number from the graphite plate may be attributed to the measurement limit of the technique or to possible secondary reactions that can occur with C, O or N for proton energies above a few MeV. Subtracting the carbon plate alpha signal as a background and correcting for the solid angle, the final alpha yield for the boron plate is *N*_α_ = (1.3 ± 0.7) × 10^8^ sr^−1^ J^−1^.

## Conclusions

An array of multiple filtered CR-39 detector plates, in combination with careful image analysis, a pit-diameter calibration for ion species and energy, and an understanding of the energy loss statistics, forms a compact and inexpensive particle spectrometer that can be easily fielded in large quantities for three-dimensional, space-resolved, multi-ion spectroscopy. The application of such a spectrometer is not limited to laser-plasma interaction experiments but can have a much broader impact. CR-39 spectrometers could be fielded in other proton-boron fusion experiments^71^ to measure alpha particle yields in strong proton and heavy ion backgrounds with high fidelity. More generally, the spectrometers can be fielded in any experiments where models of the particle spectra exist, provided a calibration for those particles has been performed.

To further increase the understanding of the uncertainties of CR-39 multi-ion spectroscopy, more advanced multivariate optimization methods such as Markov-Chain Monte-Carlo sampling^72^ may be implemented. Additionally, our current process of manually acquiring microscope images limits the effective detector area to ~ 1 mm^2^ per CR39, resulting in a small detection solid angle and in results that may still suffer from insufficient statistics. Replacing current manual methods with an automated high-throughput processing (HTP) system would reduce user-dependent uncertainty and has the potential to handle tens of thousands of samples per day. A HTP system with robot driven processing including parallel etching and microscopy can eliminate laborious error prone tasks, significantly improve statistics, data repeatability and reliability. This would further improve CR-39 ion spectroscopy during preparation, data acquisition, and analysis stages^73^.

Although the research presented in this manuscript does not claim to draw a definite approach for the analysis of CR-39 detectors so that distinctive accelerated ion species can be unambiguously distinguished and quantified, it paves the way for further optimizing the analysis of such detectors which are, for example, of fundamental importance and widely used as an alpha particle diagnostic in proton-boron fusion processes. We have found that for short etch times, pits produced by alpha particles from proton-boron fusion reactions have the same diameters as proton pits in the Landau tail. In particular, data analysis without employing an advanced pit recognition algorithm and without including the Landau tail can lead to an overestimate of the inferred alpha yields. In the data presented here, this discrepancy results in a difference of about 200 times higher inferred alpha particle yield (see Suppl. Materials). Such a result would have a significant impact on further conclusions on the viability of laser-driven proton-boron fusion. Nevertheless, the still impressive particle yields from the structured boron sample used in our experiments encourages further investigations into the viability of high-contrast, short-pulse lasers interacting with engineered targets to create advanced ion acceleration schemes, high energy density plasmas, or thermonuclear fusion conditions.

## Methods

### CR-39 etching, cleaning and microscopy

TASTRAK™ CR-39 plates by Track Analysis Systems Ltd with dimensions 20 × 20 × 1.5 mm^3^, equipped with laser-engraved consecutive numbering for identification, were purchased from Mi.am Srl, Italy. After ion exposure, they were etched to enlarge the latent tracks to the point where they can be observed with an optical microscope. The etching was performed for 30 min at 70 °C in 6.25 M NaOH solution to minimize pit overlap and to minimize the visibility of proton tracks, which appear over extended etching periods^18, 54^ and which could lead to oversaturation of the detector for longer etch times. After etching, the samples are quenched twice in DI-water and rinsed repeatedly. The samples are then stored in DI-water and rinsed individually. Afterwards they are first dipped, then rinsed with isopropanol. Finally, they are air-blown dry. The rinsing and drying steps were helpful in removing any residue from the surface, thus aiding the microscopy. The latter was performed with a Keyence VHX-7000 digital microscope, equipped with a VHX7100 fully integrated head unit. The minimum microscope resolution (Rayleigh criterion) was determined to be 0.4 µm using a commercial high-resolution test chart. During digitization, we apply manual focusing to maintain the optimal focus to within a few percent, allowing for accurate characterization of the pits. For each CR-39, 100 pictures corresponding to a size of 114 × 85 µm^2^ and a pixel size of 40.7 nm in the object plane were taken for sufficient statistics.

### Calibration measurements

Calibration measurements for pit diameter versus particle species and particle energy were performed at the tandem accelerator at the Institute for Plasma Physics (IPP) in Garching, Germany, for H, He and C ions, by using a Thomson parabola ion spectrometer fielded at the Texas Petwatt laser facility at the University of Texas in Austin, TX, USA for H and C ions, and from a ^239^Pu calibration source for He ions. Starting with the latter, the 1.5 kBq ^239^Pu calibration source emits alpha particles with 5422.43 keV. The 6-mm diameter source was placed 5.6 mm from the CR-39 detector. Two different 2 × 2 cm^2^ CR-39 samples were equipped with 6 different Al filters, in addition to the air gap, to attenuate the alpha energy between 4.7 and 1.3 MeV. The CR-39 were exposed to the alpha source for 5 min and then etched and processed as described in the main text.

A wide-range energy calibration for carbon ions was obtained from a single shot of a laser-driven ion source with a Thomson Parabola^66^, equipped with a large-area (9 × 9 cm^2^) CR-39 detector and fielded at the Texas Petawatt laser during an experiment. The laser target was a thin foil made of CH to minimize secondary ion contamination along the *q*/*m* = 0.5 trace to detect only C ions and protons. After irradiation, the plate was processed as described above. For digitization, 600 images along the *q*/*m* = 0.5 trace were taken, including the absolute position of the image on the CR-39 plate with respect to the origin of the parabolic traces. After processing with the HWHM method, the energy-dependent incidence angle per image was calculated to obtain the eccentricity via $\epsilon = 1-\sqrt{1-{b}^{2}/{a}^{2}}$, where *a* and *b* denote the half-axes of the ellipse, to compensate for the eccentricity of the pits due to the deflection in the TP and corresponding non-normal incidence on the CR-39. Without this correction we noticed a systematic offset of the measured pit diameters compared to the diameters obtained at the tandem accelerator at normal incidence for the same carbon energies. The same CR-39 plate was intended to be used to measure pit size versus energy for protons. However, no proton pits could be detected. The CR-39 plate had rectangular cutouts in regular intervals to detect the ion spectrum on an image plate underneath the CR-39. The image plate shows a clear and strong proton trace up to several tens of MeV energy. The low-energy cutoff of the instrument is at ~ 0.5 MeV for protons. This clearly demonstrates that proton pits above 0.5 MeV are too small to be detected in our configuration.

To obtain pit diameters at very low to intermediate energies, a series of calibration measurements was performed at the IPP Tandem accelerator in Garching, Germany. We used Rutherford backscattering (RBS) in a 100-nm-thin Au foil to attenuate the beam from the minimum accelerator flux rate of 10^9^ ions/cm^2^/s to the required levels for calibration. RBS leads to a slight broadening of the particle spectrum due to partial energy loss of the ions in the foil. The backscattered ion spectra were calculated with the software SIMNRA^74^. Depending on the ion energy, the energy loss was between 10 and 40%. Using the energy loss to our benefit, we obtained data down to almost 0.1 MeV. The maximum energies were 4 MeV for H, 8 MeV for He, and 10 MeV for C ions. For each ion energy, up to 20 CR-39 plates with 2 × 2 cm^2^ area were fielded simultaneously at angles between (180 ± 25)° to increase the likelihood of good irradiation statistics. Post-irradiation, the CR-39 were processed as described above.

### CSU experiment details

**Figure 9:**
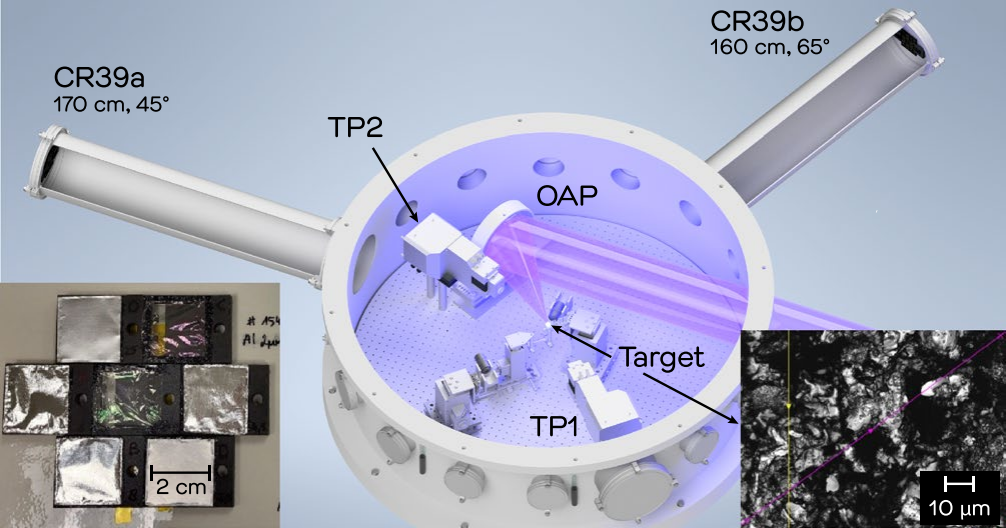
The figure illustrates the experimental setup at the ALEPH laser system, featuring the incoming laser beam, the off-axis parabolic mirror (OAP) to focus the laser pulse onto the target placed at the center of the target chamber, and the ion diagnostics that consisted of Thomson Parabola ion spectrometers (TP) and two sets of filtered CR-39 detectors. The left inset shows the configuration of CR-39 arrays in custom, 3d-printed frames (dark grey structure) equipped with filters of different thickness. The right inset shows a microscope image of a boron target surface featuring modulations from nanometers to about 10 µm.

The experiment campaign was performed at the Advanced Laser for Extreme Photonics (ALEPH) at Colorado State University (CSU). ALEPH is a frequency-doubled Ti:Sapphire laser system that can deliver up to 0.85 PW at a central wavelength of 400 nm with excellent laser contrast^75^. During this experiment the laser was focused onto boron targets using an f/2 off-axis parabolic mirror (OAP, see Fig. 9), which in this campaign delivered around 2.5 J in 88 fs within a focal spot of 1.6 µm FWHM. Analysis of the focal spot via a high-dynamic-range image reconstruction showed that the laser pulse reached an intensity of 4 × 10^20^ W/cm^2^ on target.

The targets were positioned in the laser focus using a motorized XYZ-stage. Within one experimental run up to 6 individual targets were irradiated. Boron targets were made from a commercial (Goodfellow Cambridge Ltd.), 2-mm thick, hot-pressed boron plate with a rough surface. Note that without special treatment all targets in such experiments exhibit a few-nm-thick layer of hydrocarbon impurities from CH, oil or water vapor on their surfaces. To diagnose the accelerated ions, two different particle diagnostics were fielded in the vacuum chamber. These diagnostics consisted of two Thomson Parabola (TP) ion spectrometers, one positioned along the laser propagation direction (TP1), while the second one was placed close to the OAP with an angle of ~ 35° with respect to the target normal (TP2). Additionally, two arrays of seven CR-39 solid-state nuclear track detectors with different filters were positioned at distances of ~ 1.7 m from the interaction point at two different angles with respect to the target normal (45° for CR-39a & 65° for CR39b). Custom, 3D-printed frames allowed reproducible placement of the 2 × 2 cm^2^ CR-39 via slots on the side of the frames. Each frame was covered with one of the following filters: 1 μm Mylar, or aluminum of 2 μm, 4.5 μm, 8 μm, 12.5 μm and 25 μm thickness. The central CR-39 served as a witness sample and was etched immediately following a shot series to verify that the particle flux was not saturating the detectors.

### Supplementary Information

Supplementary Information.

## Data Availability

The data generated in this study are available from Marvel Fusion (info@marvelfusion.com) upon reasonable request.
