# Supplementary material for: Differentiating multi-MeV, multi-ion spectra with CR-39 solid-state nuclear track detectors
Source: Sci Rep. 2023 Oct 24;13:18155. doi: 10.1038/s41598-023-45208-x (PMC10598230; doi:10.1038/s41598-023-45208-x)
Supplement: Supplementary file 1 — Supplementary Information. [file 41598_2023_45208_MOESM1_ESM.pdf]

Supplementary Materials for: Differentiating multi-MeV, multi-ion spectra with CR-39 solid-state nuclear track detectors

Authors: M.S. Schollmeier*[,1], J.J. Bekx[1], J. Hartmann[1], E. Schork[1], M. Speicher[1], A.F. Brodersen[1], A. Fazzini[1], P. Fischer[1], E. Gaul[1], B. Gonzalez-Izquierdo[1], M.M. Günther[1], A.K. Härle[1], R. Hollinger[2], K. Kenney[1], J. Park[2], D.E. Rivas[1], V. Scutelnic[1], Z. Shpilman[2], S. Wang[2], J.J. Rocca[2,3], and G. Korn[1]

Affiliations:
[1]Marvel Fusion GmbH, Theresienhöhe 12, 80339 Munich, Germany
[2]Electrical and Computer Engineering Department, Colorado State University, Fort Collins, Colorado 80523, USA
[3]Physics Department, Colorado State University, Fort Collins, Colorado 80523, USA

1. More details about the HWHM method and thresholding

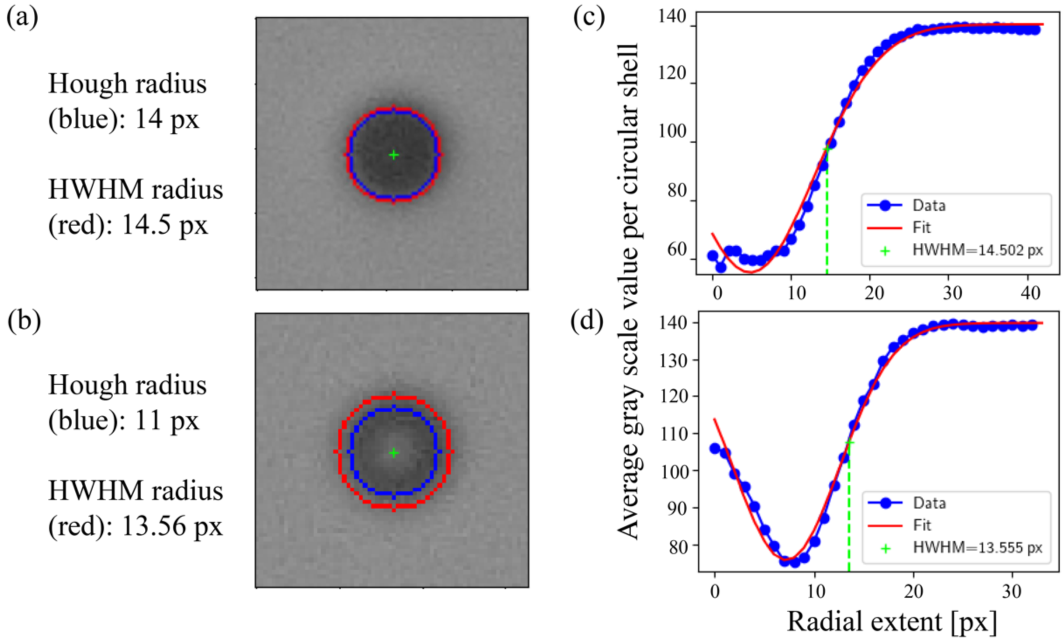

Figure S1: A typical example of a prominent pit (a) and donut (b), with their centers depicted by the green plusses, the results from the Hough transform in blue, and the corrected HWHM radii shown in red. The lax and strict Hough thresholds were g_{lax} = 132 and g_{strict} = 100, respectively. (c and d): The corresponding average grayscale value per cylindrical shell as a function of their radial extent (blue dots), with the corresponding fit with Eq. (1) of the main manuscript shown in red. The radius calculated by the HWHM method is also shown in green.

An example exhibiting the typical behavior that justifies the modeling of the grayscale values of an imaged pit with an upside-down Gaussian is shown Figure S1. Figure S1a shows an example of a typical prominent pit, with its center shown as a green plus. In Figure S1b, we plot the result from the radial shell averaging and the subsequent fitting with Eq. (1) of the main manuscript. Note that the noisiest data points are found at the lowest values for the

radial extent, that the minimum of the fit is found at a $\mu > 0$, and that the background can be seen by the plateau region.
Figure S1c and S1d were added to illustrate that the HWHM-method also works for pits whose centers do not constitute the darkest part of the pit and are more reminiscent of a donut-like shape, which have been seen to occur in previous works [1, 2]. For these cases, the darkest part lies in the center of the darker ring, which is clearly seen in the bottom-left figure. The resulting fit still works, but with a larger value of $\mu > 0$, as is seen in the bottom-right figure.

One might argue that setting the HWHM as the radius is in and of itself a user input and again prone to bias. One could have chosen to take the radius not at half the maximum, but rather at ¾ -th of ¼ -th of the maximum. The effects of setting the radius to ¾-th or ¼-th of the maximum are shown in the left and right plots of Figure S2, respectively. Consistent statistical behavior of the histograms is recovered so long as a single choice is adhered to.

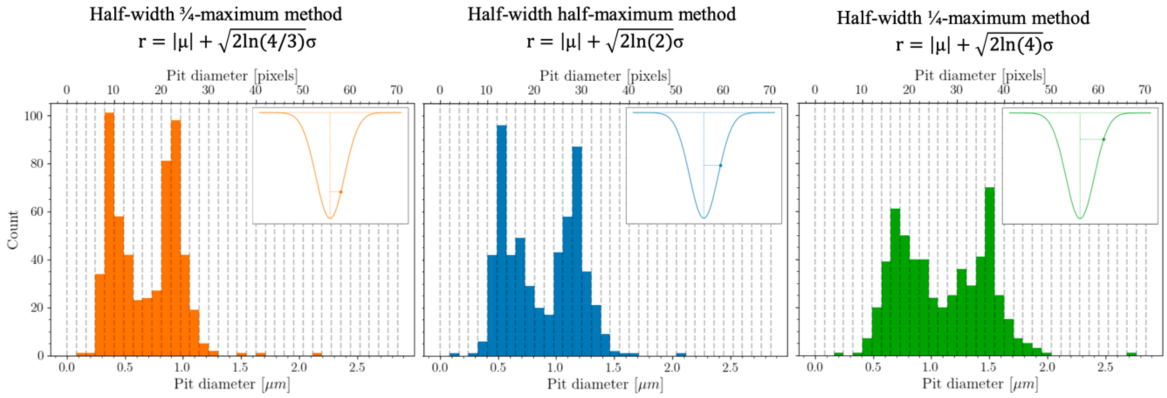

Figure S2: The effects of setting the radius to ¾-th or ¼-th of the maximum are shown in the left and right plots, respectively. Though two prominent peaks corresponding to the two particle species remain, the values at which these peaks arise alter. The point remains however that, no matter the choice of radius made, the same relative internally consistent method to assign the radii is used. This removes the thresholding cut-off artifact. Consistent statistical behavior of the histograms is recovered so long as a single choice is adhered to.

2. Measured energy loss distributions

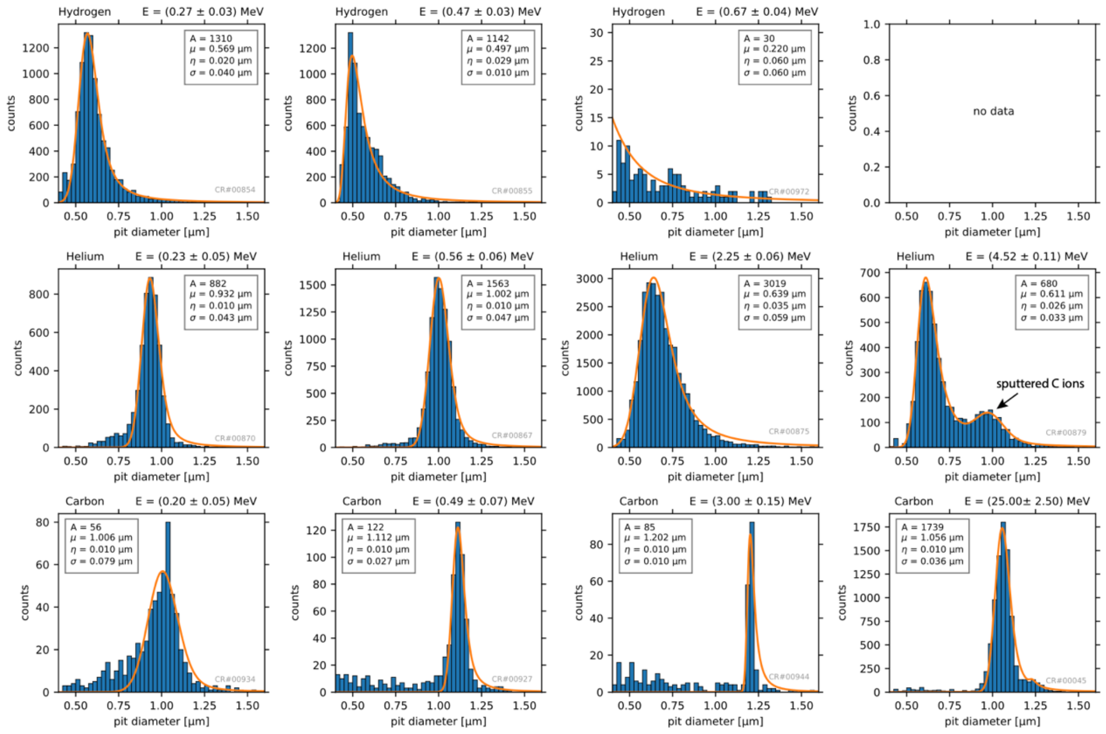

Figure S3: Measured pit size distributions for particle irradiation with a narrow energy distribution. The top row shows examples for hydrogen, the middle row for helium, and the bottom row for carbon ions. From left to right, the columns are sorted by particle energy in ascending order. The orange curves plot the best-fitting Langau distributions, the fit parameters are noted in the boxes in the top right or left corners of each subplot. The titles of each plot show the projectile average energy and energy interval. The data set for protons is limited; above an energy of about 0.5 MeV the proton pits are too small to be detected with our setup. Proton data above this energy were excluded from further analysis.

Figure S3 shows some representative pit size distributions after irradiation with mono-energetic ions. The depicted histograms are of similar ion energies for the three ion species to better visualize the differences. Most data were taken from the ion accelerator calibration (see Methods), except for carbon ions above 2 MeV which were taken from the TP. It was verified that the two data sets from IPP and TP result in comparable histograms at similar energies.

The middle row shows data for He ions. The Langau distribution matches the measured data very well. Its widths are on the order of 0.4-0.6 μm. For energies above 3 MeV an additional peak occurs in the histogram (see the right-most panel in the middle row for an example). Here, a fit was performed with a Langau plus an additional, shifted Gaussian distribution to remove the influence of the secondary peak on the σ_L determination. This secondary peak is attributed to surface carbon or oxygen ions being sputtered by the fast He ions in the RBS setup. The peak location at around 1 μm matches the peak position of low-energy carbons. Note that the RBS measurements were performed without an additional filter in front of the CR-39 to stop low-energy particles from sputtering events. Future calibration runs will include a protective foil in front of the CR-39.

The energy-dependent behavior of the carbon pit size distributions is shown in the bottom row of the figure. Interestingly, going from low to high energy the distribution starts relatively broad, then becomes very narrow at about 1-3 MeV, and then broadens again. The narrowly peaked distribution was observed to be identical in both the IPP and the TP data sets, therefore we can rule out any systematic error in the experiment. An explanation for such scaling is given in the main manuscript.

We summarize the Landau distribution widths in Table S1. In the table, σ_L and η_L give the ranges of widths of Landau (σ_L) and Gaussian (η_L) encountered during the analysis of the calibration data. The last column shows the average value, which is also used in the forward-fitting model. Interestingly, the absolute width of the distribution decreases with increasing particle mass. Protons create the broadest distributions with almost 60 nm width, whereas carbons result in much narrower distribution of about 25 nm width. The distribution for Helium ions is in-between these two data sets, which is to be expected.

Table S1: Landau pit size distribution widths for H, He, and C. Columns σ_L and η_L show the ranges of widths of Landau (σ_L) and Gaussian (η_L) encountered during the analysis of the calibration data. The last column shows the average value, which is also used in the forward-fitting model.

ion	σ_L [µm]	η_L [µm]	$\sigma_{eff.}$ [µm]
H	0.040 – 0.060	0.020 – 0.030	0.060
He	0.030 – 0.060	0.010 – 0.035	0.050
C	0.008 – 0.050	0.010	0.025

3. TRIM simulations of energy loss in CR-39

We have performed ion tracking simulations with TRIM as described in the main manuscript. Specifically, a total of 2,000 monoenergetic carbon ions were tracked through a 2-μm thick layer of CR-39 followed by a 5-μm thick Al absorber layer. Both, the CR-39 and Al materials are available in the SRIM Compound Library. A few convergence test cases were run with 20,000 ions, no significant deviation of the results was found. In TRIM, the easiest approach to simulate radiation damage is to calculate the displacements per atom (dpa) using the depth-dependent vacancies produced by incident ion and recoils, which TRIM summarizes in a dedicated file for all ions of one species. However, a simple summary is not of interest for our purposes. Additionally, the SRIM processor used to generate this file overestimates the vacancy production up to a factor of four [3]. Instead, we set the software to store all particle collisions in a separate file. Post-simulation, this file was analyzed using a custom Python script to gather the distribution of vacancies for each ion in a specified detector depth. Assuming a direct proportionality between the number of vacancies and the resulting pit diameter in CR-39, we subsequently plot a histogram of the number of produced

vacancies per ion vs. their occurrence. The results are then fitted with a Landau distribution to retrieve the most probable vacancy number μ_{TRIM} and the width σ_{TRIM} of the distribution.

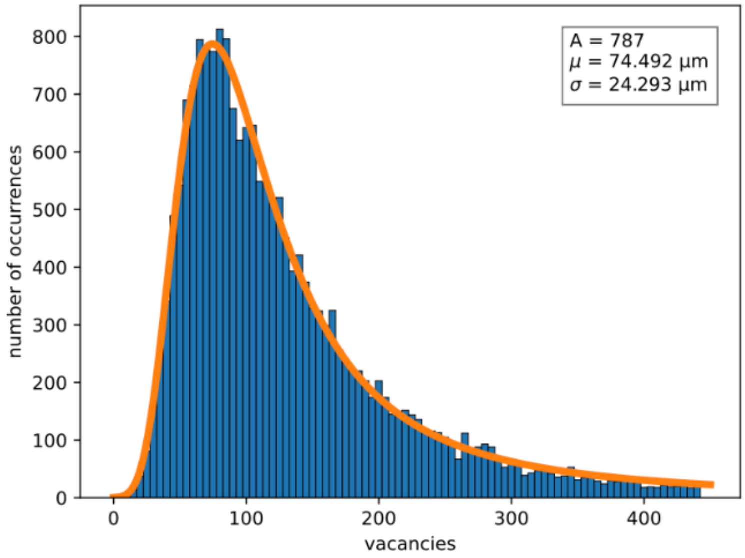

Figure S4: TRIM simulation results of vacancy production for 20,000 carbon ions with 600 keV initial energy, propagating into CR39, plotted for an integrated penetration volume from 0 to 1 μm depth. The distribution closely resembles a Landau distribution (orange curve) with an amplitude A = 787, most probable vacancy production of $\mu_{TRIM} \approx 74.5$, and width $\sigma_{TRIM} \approx 4.3$.

Figure S4 shows an example histogram for 600 keV carbons. The stopping range of 1.5 μm for this energy is beyond the analyzed volume. A Landau fit to the data allows us to extract the most probable vacancy production μ_{TRIM} and width σ_{TRIM} to obtain the ratio σ_{TRIM}/μ_{TRIM} for comparison to our measurements. This procedure was repeated for several carbon energies from 0.6 to 60 MeV. The results are shown as the black data in Figure 5 in the main manuscript. We observe in the simulation data that, going from low to high energy, the μ of the distribution is moving towards fewer vacancies, meaning that faster ions produce less damage in the first μm of the material. However, the width of the distribution is also becoming narrower because higher energy ions generate fewer high-damage events when their energy is away from the Bragg peak. At around 4 MeV, the most probable vacancy production is less than one. In the measurement this corresponds to zero track formation, which we cannot detect. Since a track is observed, the most probable value was manually set to a value near 1 to include this in our TRIM analysis of vacancy production. Without clamping the most probable value to near 1, no meaningful Landau distribution fit could be performed. Since μ stays near 1 for energies higher than 4 MeV but the vacancy production becomes less with increasing energy, the apparent width of the distribution, normalized to the most probable value, becomes smaller as observed in the experiment.

4. Test of the forward fitting method

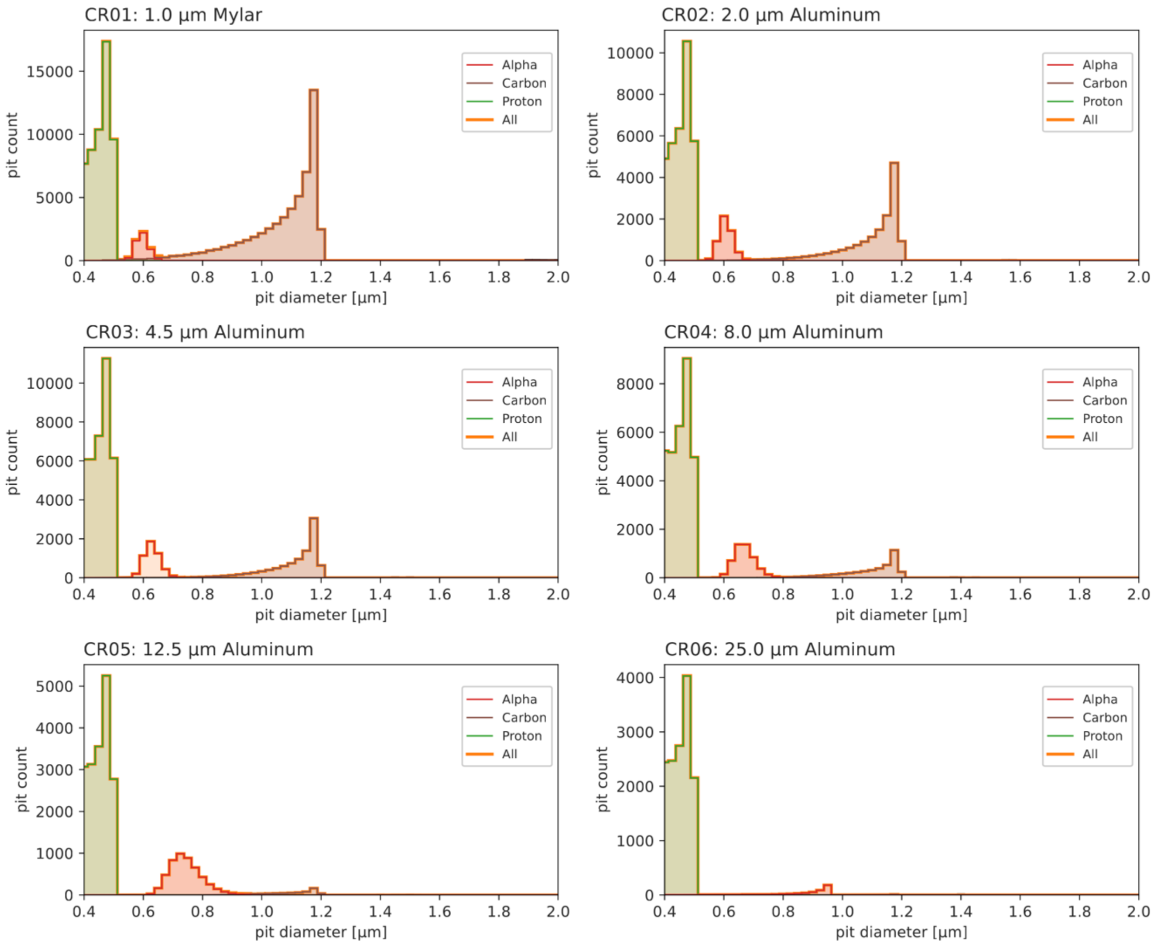

Figure S5: Calculated pit distribution histograms for an array of six filtered CR-39 detectors, ordered by filter thickness from top left to bottom right, for H, He and C *<u>without including</u>* *energy loss probability distributions (Landau distributions). The title of each sub panel specifies the filter material and filter thickness.*

For an illustration of the forward fitting method, Figure S5 shows calculated histograms for an array of six filtered CR39 detectors. The calculation assumed a proton spectrum following Eq. (4) in the main manuscript with N_0 = 5 × 10^5 and $k_B T$ = 5 MeV with a maximum (cutoff) energy of 10 MeV. Since the calibration curve for protons peaks at about 0.5 μm diameter, the maximum pit diameter for protons is also near 0.5 μm. Higher or lower proton energies generate smaller pits. The minimum diameter used in our analysis is 0.4 μm, because our microscope cannot resolve features smaller than this value.

The brown curve plots the results for carbon ions with a Boltzmann distribution, Eq. (3), with N_0 = 1 × 10^5 and $k_B T$ = 2 MeV and a cutoff energy of 100 MeV. Similar to the proton data, the pit size distribution has its peak at the maximum of the calibration curve, with a sudden drop towards larger pit diameters and a long tail towards smaller pit diameters, whose slope depends on the temperature of the ion spectrum.

Lastly, in red we have plotted a Gaussian distribution of 5,000 alpha particles with a mean energy of 5 MeV and a FWHM of 1 MeV. The stopping range of 5 MeV alphas in aluminum is 21.4 μm according to SRIM, which is why CR06 shows a small signal from the tail of the Gaussian only.

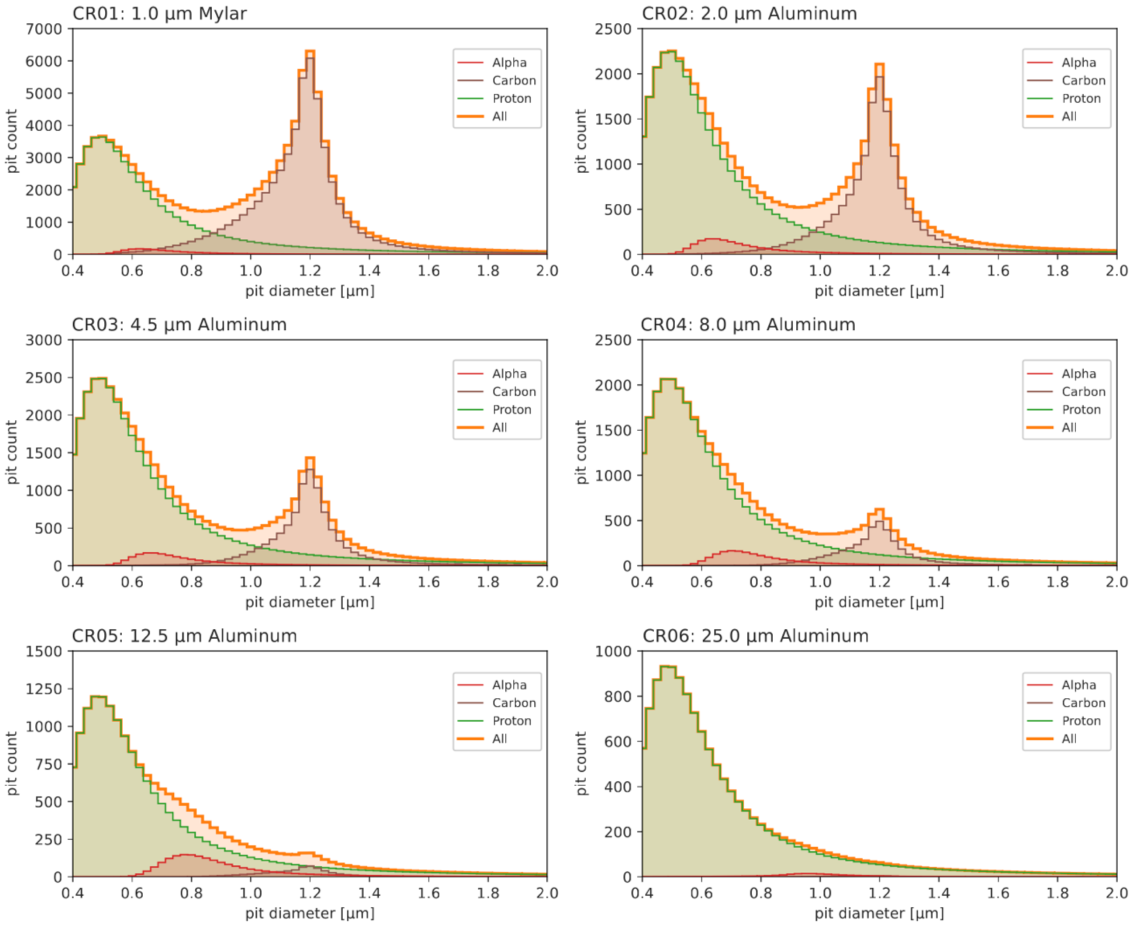

Figure S6: Calculated pit distribution for H, He and C <u>including</u> energy loss probability distributions (Landau distributions). The figure is otherwise identical to Figure S5.

The synthetic histograms were calculated without considering a realistic energy loss probability distribution for thin detectors (Langau distributions). If this could be neglected, the three different particle species would generate well-separated pit size distributions without any significant overlap even for exponential particle spectra. In this case, alpha particles would for example be found for pit diameters between 0.6 and 0.8 μm, carbons at around 1-1.2 μm, and protons for 0.5 μm and below, which would result in an almost trivial data analysis and particle counting for a laser experiment. However, the energy loss probability distribution for each particle species is of utmost importance for the correct interpretation of measured signals. This becomes obvious when looking at Figure S6, for which we have used the exact same input spectra as for Figure S5, but switched on the convolution with the Landau function. Specifically, we have used a σ_L = 0.06 μm for protons, σ_L = 0.03 μm for alphas, and σ_L = 0.02 μm for carbons. The most striking change is the strong broadening of the proton pit distribution, which now extends to well above 1 μm. Similarly,

the carbon distribution becomes almost symmetric as the convolution of the Landau distribution creates a significant right tail. The initially Gaussian alpha particle distribution does not significantly change its shape. However, because of the strong proton tail due to the Landau distribution, the contribution of the alpha particles to the total histogram (orange line) is nearly negligible except for the 12.5 μm Al filter where it creates a small hump. Identifying alpha particles in such a pit-size distribution becomes much more challenging and requires sufficient statistics and careful analysis to distinguish a potential alpha particle contribution to the right tail of the proton pit distribution.

To test the method for its reliability in obtaining particle spectra, and for its sensitivity to infer alpha particles in a mixed-ion irradiation field, we create artificial histograms similar to Figure S6 but add random Gaussian noise to the data. The variance of the noise was chosen to be 15 % of the average value of the counts for each CR-39. This noise level is comparable to typical experiment data.

Figure S7 shows the resulting histograms for six differently filtered CR-39, assuming the same filters as in the previous section. The artificial data (blue bars) are calculated assuming protons with N_0 = 5 × 10^5, $k_B T$ = 2 MeV and a spectrum according to eq. (4), N_0 = 3 × 10^4 carbon ions with $k_B T$ = 6 MeV and a spectrum according to Eq. (3), and a small population of N_0 = 1,000 alpha particles with Gaussian distribution centered at E_0 = 4 MeV and 1 MeV FWHM. The alpha particle count corresponds to 0.19 % of the total particle count.

To perform a numerical fit to the data, methods such as least-squares regression can be employed. However, due to the noise in the data a simple regression algorithm can run into a local minimum and miss the global one. An easy-to-implement, but computationally expensive fitting optimization method is Monte Carlo sampling, where a large set of randomly selected input parameters are sampled within user-selected boundaries. Specifically, to determine the range of best-matching spectra for the noisy, artificial data, we draw 2 × 10^5 samples for N_0 and $k_B T$ (E_0 for the alphas, respectively) for the three particles to find the global minimum. Table S2 specifies the true parameters, the minimum (maximum) parameter MC_{min} (MC_{max}) for the search, and the optimum parameter MC_{opt} found by the Monte Carlo scan. The optimum values are close to the true values, with a deviation corresponding to the added noise level of the artificial data. Note that the number of samples is probably not sufficient to sample fully adequately from the large parameter ranges. The number of MC samples is currently limited by the implementation of the software, which was written in single-threaded Python. The MC scan took about 9 hours using a 2021 MacBook Pro with Apple M1 Pro chip (0.6 - 3.22 GHz). We expect that significant speedups can be obtained by switching to parallel execution and a more efficient programming language such as, for example, C or C++.

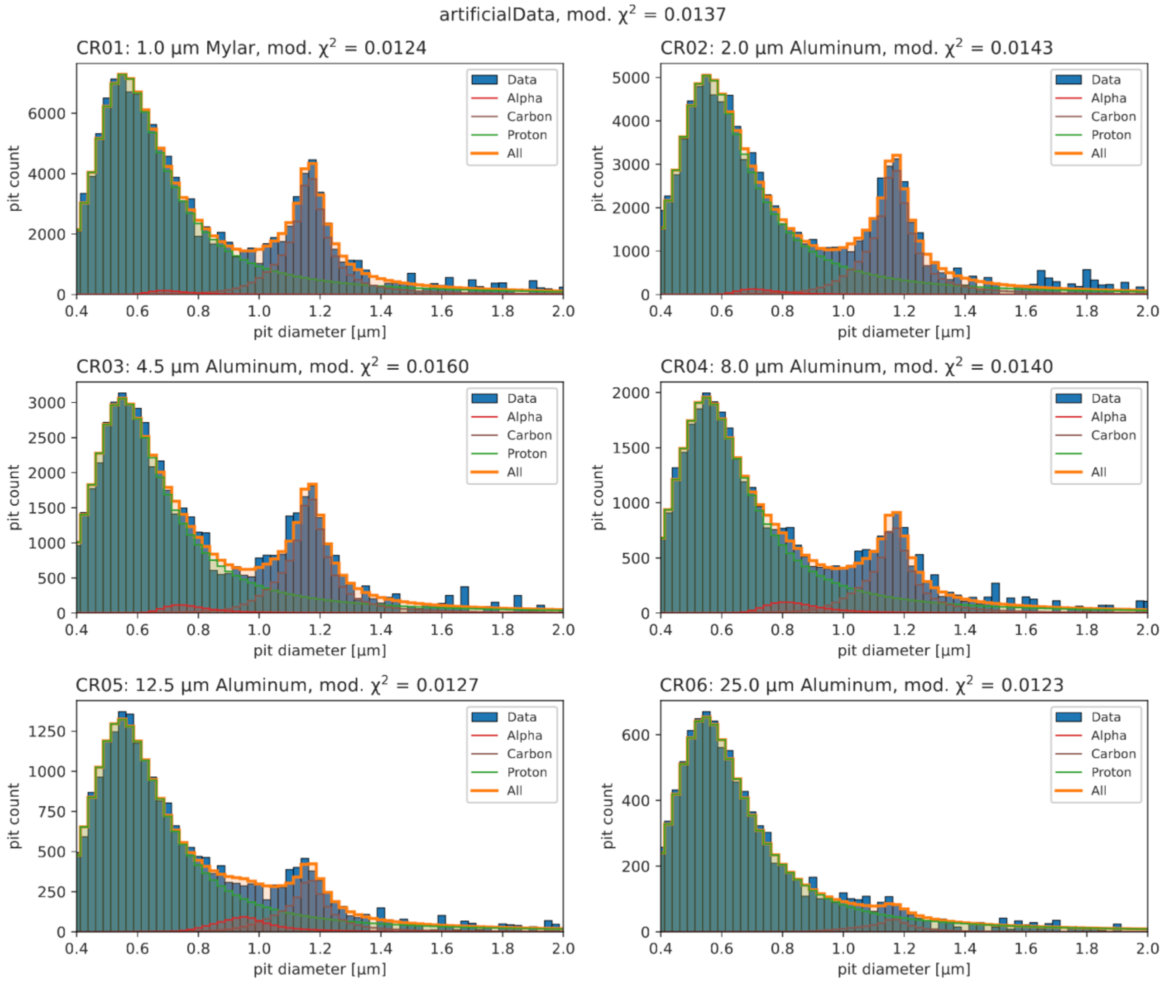

Figure S7: Artificial data for testing the forward fitting method. The data (blue bars) are calculated for six differently filtered CR-39 with filters specified in the title of each panel, irradiated by protons, carbons and a small fraction of alpha particles. The shaded area plots show the forward-fitted data.

Table S2: Comparison of true and fitted parameters for the artificial histogram. Monte Carlo sampling was performed within the parameter ranges specified by $MC_{min/max}$. The optimum values are close to the true values, with a deviation corresponding to the added noise level of the artificial data.

parameter	true value	MC_{min}	MC_{max}	MC_{opt}
Protons:				
N_0	5×10^5	4.9×10^5	5.1×10^5	5.02542×10^5
k_BT	2	0	4	2.14
Carbons:				
N_0	3×10^4	2.5×10^4	3.5×10^4	3.0404×10^4
k_BT	6	4	8	5.95
Alphas:				
N_0	1000	0	2000	1192
E_0	4	0	8	4.16

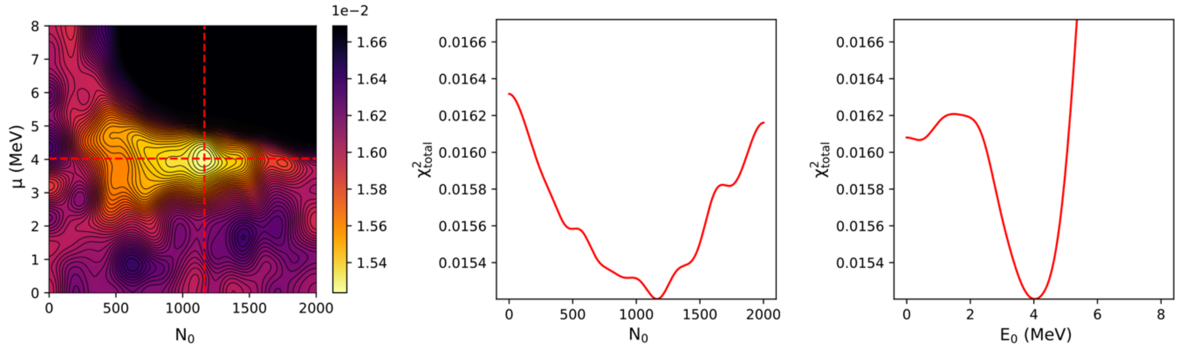

Figure S8: Sensitivity scan for the artificial data. The left panel shows a global minimum near the true values, which are N_0 = 1000 and μ = 4 MeV. The center and right panels show one-dimensional lineouts of χ^2 vs. N_0 and E_0 at the optimum locations. The color gradient, as well as the slope of the line-outs, shows that the method is much more sensitive to changes of E_0 than N_0, as described in the main manuscript.

To obtain an error estimate of the inferred alpha particle number, Figure S8 plots χ^2 vs. N_0 and total k_BT for the alpha particles. We use such a sensitivity analysis to infer error of the fit method due to the noisy input. Since the overall MC scan sampled over a relatively large range for all input parameters, the data had to filtered first by taking the optimum values for protons and carbons and extracting only those parameters that are within ±15 % of the optimum values. This resulted in about 13,000 samples. Of those data, a two-dimensional map was created where the two axes are the N_0 and E_0 of the alpha particles. From lineouts through the optimum value in N_0 and E_0 direction, we infer the sensitivity of the method and define the FWHM of the line-out as the error. The inference of the total number of alpha particles results in about 50% error. However, it is encouraging that the method can infer the true alpha particle number to a reasonable degree considering that the overall noise was set to 15 %, which is about 100 times above the alpha contribution (0.19 %, see above) to the overall particle number.

The total error of the analysis is mainly determined by the particle counting statistics ($\sqrt{N}$), the variance of the calibration data (see main text), the error of the best-fitting width of the Landau distribution, the error of the etching, image acquisition error, and the error of the fitting method itself. For the artificial data, the calibration data or Landau widths were not altered between data creation and fitting, hence their error is zero. Etching errors, image acquisition errors, etc., are also not relevant for the example. The remaining errors for the alpha particle number N_α are due to particle statistics, which is $\sqrt{1192}$ = 34.5, and the error of the fitting method, which we estimate to be about 50 % (596 particles). This leads to an overall error of ΔN_α = ± $(34.5^2 + 596^2)^{1/2}$ = 597, or $\Delta N_\alpha / N_\alpha$ ≈ ±50 %. The error for the mean energy can be obtained by the sensitivity scan as ΔE_0 ≈ ±1 MeV, or $\Delta E_0 / E_0$ ≈ ±25 %.

A better error analysis method would be to perform a detailed uncertainty quantification (UQ) study or Markov Chain Monte Carlo (MCMC). UQ involves quantifying and propagating

uncertainties in the inputs and outputs of a model to provide more accurate and reliable predictions, including all error variances from calibration errors. MCMC enables the exploration of high-dimensional parameter spaces and estimation of posterior distributions. By combining MCMC with Bayesian inference, UQ can be performed by incorporating prior knowledge and updating it with observed data to obtain the posterior distribution of model parameters. This posterior distribution provides information about the uncertainties associated with the estimated parameters. A dedicated implementation of MCMC is currently ongoing, the results of which will be described in a future report.

5. Comparison of alpha yields using our improved method vs. a simplistic pit diameter assignment

This section compares the new CR-39 analysis method described in this report to the previous status quo. Specifically, we demonstrate that image analysis using simple thresholding, in combination with an incomplete calibration (e.g., without considering C, B, or O ions) [2, 4-9] and particularly without considering energy loss statistics, leads to inferred alpha particle numbers that are incorrect by several orders of magnitude and particle energies that are also incorrect by a few MeV, due to incomplete understanding of how the CR-39 operates as a particle detector.

We use the *exact same set of microscope images* from CR-39b described in the main manuscript for a demonstration. We assume that the CR-39 detector was calibrated for alpha particles with energies of a few MeV maximum energy down to a few hundred keV, e.g., as one can adjust using a radioactive source with absorber foils [2, 5, 7, 10]. Such an incomplete data set could look like Figure S9, where we have plotted the proton and alpha calibration from this work. Additionally, an alpha 'calibration range' [5] mimicking a radioactive source calibration has been highlighted, corresponding to alpha particle energies from 5.75 MeV to 1 MeV. Those energies correspond to diameters from 0.57 μm to 0.92 μm. This diameter range starts just above the maximum proton pit diameter and ends close to the maximum alpha pit diameter.

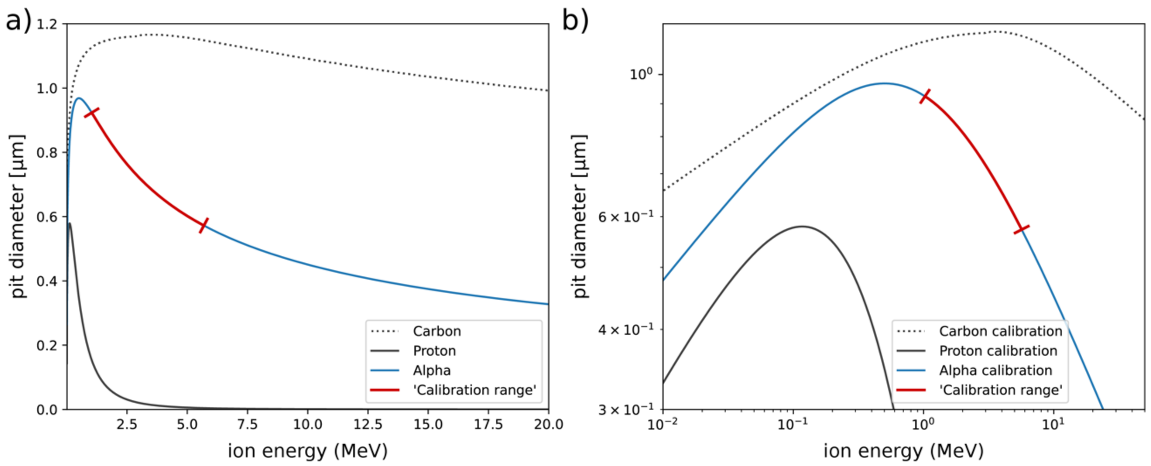

Figure S9: Comparison of an alpha 'calibration range' similarly defined as in the literature [2, 5, 7, 10] to the full curves obtained in our work. a) linear scale, b) logarithmic scale.

Next, we analyze the microscope image data set using a simple threshold pit recognition method to find locations and diameter of the pits. The result for the CR-39 behind the 4.5 μm Al filter is shown Figure S10a. Note that the threshold method results in a histogram with four peaks. This histogram is in stark contrast to the top right histogram shown in Figure 6 in the main manuscript, which was obtained using the HWHM method. Again, both histograms have the exact same microscope image data set as underlying basis. The total particle count for both histograms is also exactly the same with 70,793 recognized pits.

The alpha particle calibration range is highlighted in red, forming the second peak in the histogram. This histogram leads to an interesting, though incorrect, interpretation of the data assuming that the pit size is proportional to ion mass: the first peak is formed by protons, the second peak is from alpha particles, the third peak are borons and the fourth peak is formed by carbons or oxygens from the surface contamination. There may even be a fifth population of ions at around 1.6 μm diameter. However, as we have shown in the main manuscript, those peaks are artifacts caused by rounding errors of the threshold algorithm.

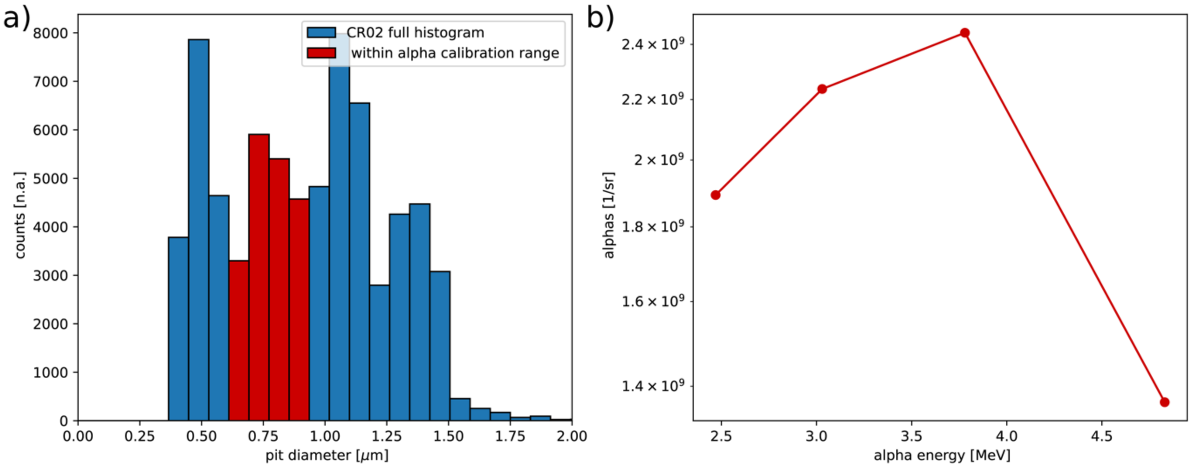

Figure S10: Inferring an alpha spectrum from the pit size distribution in CR-39 using the threshold and calibration range method. a) histogram of pit sizes with those pits within the 'alpha calibration range' highlighted in red. b) Assigning alpha particle energies for each bin, then correcting for filter transmission and for solid angle results in a peaked spectrum at about 4 MeV.

Ignoring this fact, an alpha particle spectrum can be built by assigning an alpha particle energy to each diameter of the highlighted bars via the 'calibration range', subsequently correcting for alpha particle energy loss due to the filter, and correcting for solid angle, to finally arrive at the alpha particle yield vs. energy plotted in the Figure S10b. Due to the peak in the histogram, the spectrum is peaked as well.

This procedure is then repeated for all filtered CR39 to build the complete spectrum by correcting for the energy loss in the filters. In the overlapping regions we take the average alpha particle yield. The resulting final spectrum is shown in Figure S11a. The spectrum peaks at about 4 MeV, matching the expectation for alpha particles from proton-boron fusion (see,

e.g., Fig. 1 in Spraker *et al*. [11]). However, the peak appears at this energy due to the chosen calibration range and filter combination, and not due to a physical process.

Integrating the spectrum results in a total alpha particle number of about 10^{11}/sr. Assuming isotropic emission, this corresponds to a total alpha particle energy of ~0.5 J for a laser energy input of 3 J, which would be about 15% conversion efficiency of laser energy into proton-boron fusion alpha particles.

Finally, Figure S11b compares the alpha particle spectrum from the simplistic method to the spectrum obtained via the method developed in this report. The total alpha particle yield using our improved method, as discussed in the main manuscript, is N_α = (5 ± 3) × 10^8 sr^{-1}. This yield is about 200 times lower than the yield from the simplistic method.

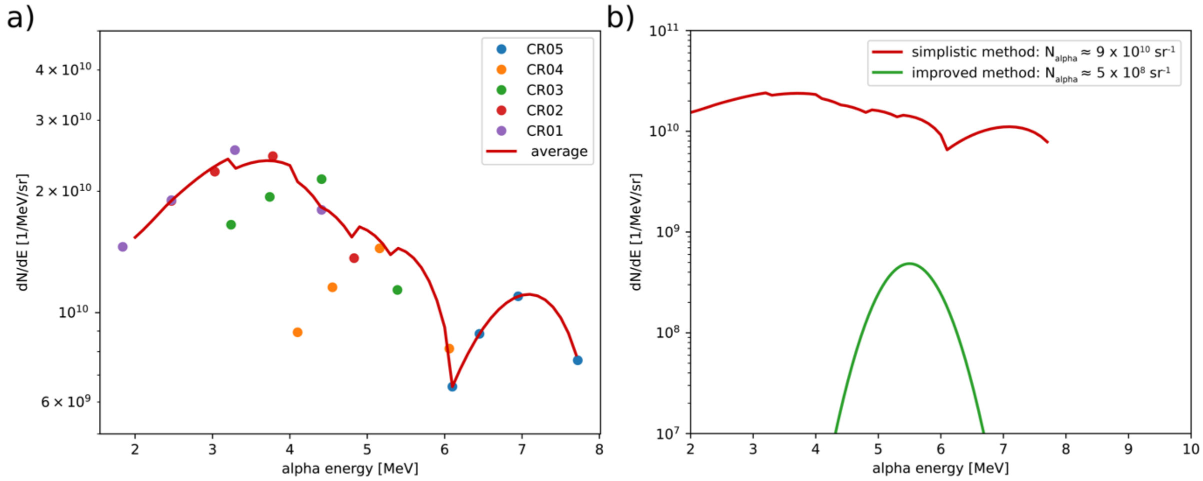

Figure S11: Average spectrum using the simplistic thresholding and calibration range method for all filtered CR-39 (red line in a and b) vs. the spectrum derived using the method developed in this report (green line in b).

6. References

[1] C. Baccou *et al.*, "CR-39 track detector calibration for H, He, and C ions from 0.1-0.5 MeV up to 5 MeV for laser-induced nuclear fusion product identification," *Review of Scientific Instruments,* vol. 86, no. 8, p. 083307, 2015, doi: 10.1063/1.4927684.

[2] D. Margarone *et al.*, "Generation of α-Particle Beams With a Multi-kJ, Peta-Watt Class Laser System," *Frontiers in Physics,* vol. 8, 2020, doi: 10.3389/fphy.2020.00343.

[3] S. Agarwal, Y. Lin, C. Li, R. E. Stoller, and S. J. Zinkle, "On the use of SRIM for calculating vacancy production: Quick calculation and full-cascade options," *Nuclear Instruments and Methods in Physics Research Section B: Beam Interactions with Materials and Atoms,* vol. 503, pp. 11-29, 2021, doi: 10.1016/j.nimb.2021.06.018.

[4] V. S. Belyaev *et al.*, "Observation of neutronless fusion reactions in picosecond laser plasmas," (in eng), *Phys Rev E Stat Nonlin Soft Matter Phys,* vol. 72, no. 2 Pt 2, p. 026406, Aug 2005, doi: 10.1103/PhysRevE.72.026406.

[5] J. Bonvalet *et al.*, "Energetic alpha-particle sources produced through proton-boron reactions by high-energy high-intensity laser beams," *Phys Rev E,* vol. 103, no. 5-1, p. 053202, May 2021, doi: 10.1103/PhysRevE.103.053202.

[6] L. Giuffrida *et al.*, "High-current stream of energetic alpha particles from laser-driven proton-boron fusion," *Phys Rev E,* vol. 101, no. 1-1, p. 013204, Jan 2020, doi: 10.1103/PhysRevE.101.013204.

[7] D. Margarone *et al.*, "In-Target Proton–Boron Nuclear Fusion Using a PW-Class Laser," *Applied Sciences,* vol. 12, no. 3, 2022, doi: 10.3390/app12031444.

[8] D. Margarone *et al.*, "Laser-driven proton acceleration enhancement by nanostructured foils," *Phys Rev Lett,* vol. 109, no. 23, p. 234801, Dec 7 2012, doi: 10.1103/PhysRevLett.109.234801.

[9] A. Picciotto *et al.*, "Boron-Proton Nuclear-Fusion Enhancement Induced in Boron-Doped Silicon Targets by Low-Contrast Pulsed Laser," *Physical Review X,* vol. 4, no. 3, 2014, doi: 10.1103/PhysRevX.4.031030.

[10] T. A. Mehlhorn *et al.*, "Path to Increasing p-B11 Reactivity via ps and ns Lasers," *Laser and Particle Beams,* vol. 2022, pp. 1-16, 2022, doi: 10.1155/2022/2355629.

[11] M. C. Spraker *et al.*, "The 11B(p, α)8Be $\rightarrow \alpha + \alpha$ and the 11B(α, α)11B Reactions at Energies Below 5.4 MeV," *Journal of Fusion Energy,* vol. 31, no. 4, pp. 357-367, 2011, doi: 10.1007/s10894-011-9473-5.
